# Melatonin: A potential nighttime guardian against Alzheimer’s

**DOI:** 10.1038/s41380-024-02691-6

**Published:** 2024-08-11

**Authors:** Zefan Zhang, Pei Xue, Barbara B. Bendlin, Henrik Zetterberg, Fernanda De Felice, Xiao Tan, Christian Benedict

**Affiliations:** 1 Department of Big Data in Health Science, Zhejiang University School of Public Health and Sir Run Run Shaw Hospital, Zhejiang University School of Medicine, Hangzhou, China; 2 The Key Laboratory of Intelligent Preventive Medicine of Zhejiang Province, Hangzhou, China; 3 Department of Pharmaceutical Biosciences, Uppsala University, Uppsala, Sweden; 4 School of Medicine and Public Health, University of Wisconsin, Madison, WI USA; 5 Wisconsin Alzheimer’s Disease Research Center, Madison, WI USA; 6 Wisconsin Alzheimer’s Institute, Madison, WI USA; 7 Department of Psychiatry and Neurochemistry, Institute of Neuroscience and Physiology, the Sahlgrenska Academy at the University of Gothenburg, Mölndal, Sweden; 8 Clinical Neurochemistry Laboratory, Sahlgrenska University Hospital, Mölndal, Sweden; 9 Department of Neurodegenerative Disease, UCL Institute of Neurology, Queen Square, London, UK; 10 UK Dementia Research Institute at UCL, London, UK; 11 Hong Kong Center for Neurodegenerative Diseases, Clear Water Bay, Hong Kong China; 12 Wisconsin Alzheimer’s Disease Research Center, School of Medicine and Public Health, University of Wisconsin, University of Wisconsin-Madison, Madison, WI USA; 13 Centre for Neurosciences Studies, Departments of Biomedical and Molecular Sciences, and Psychiatry, Queen’s University, Kingston, ON K7L 3N6 Canada; 14 D’Or Institute for Research and Education, Rio de Janeiro RJ, 22281-100 Brazil; 15 Institute of Medical Biochemistry Leopoldo de Meis, Federal University of Rio de Janeiro, 21941-902 Rio de Janeiro RJ, Brazil; 16 Department of Clinical Neuroscience, Karolinska Institutet, Stockholm, Sweden

**Keywords:** Diseases, Neuroscience

## Abstract

In the context of the escalating global health challenge posed by Alzheimer’s disease (AD), this comprehensive review considers the potential of melatonin in both preventive and therapeutic capacities. As a naturally occurring hormone and robust antioxidant, accumulating evidence suggests melatonin is a compelling candidate to consider in the context of AD-related pathologies. The review considers several mechanisms, including potential effects on amyloid-beta and pathologic tau burden, antioxidant defense, immune modulation, and regulation of circadian rhythms. Despite its promise, several gaps need to be addressed prior to clinical translation. These include conducting additional randomized clinical trials in patients with or at risk for AD dementia, determining optimal dosage and timing, and further determining potential side effects, particularly of long-term use. This review consolidates existing knowledge, identifies gaps, and suggests directions for future research to better understand the potential of melatonin for neuroprotection and disease mitigation within the landscape of AD.

## Introduction

Alzheimer’s disease (AD) is a chronic, neurodegenerative disease and the leading cause of dementia, affecting more than 50 million people worldwide. This number is projected to be greater than 150 million by 2050 [1]. Characterized by accumulation of amyloid-beta (Aβ) plaque, neurofibrillary tangles (NFTs), and neurodegeneration, sporadic AD is understood to be the result of multiple genetic factors, as well as interactions between genes and environmental factors. See Box 1 for the definition of sporadic AD and how it differs from the familial form of AD.

Among potentially modifiable risk factors, sleep disorders, including insomnia and obstructive sleep apnea, and shortened sleep duration, have been linked to AD [2–13]. Several processes have been proposed to elucidate the mechanistic role of sleep in the context of AD. These include the clearance of potentially detrimental metabolites and protein fragments, such as soluble Aβ, from the brain [14], influencing tau protein dynamics [8], supporting the function of the blood-brain barrier (BBB) [15], maintaining synaptic integrity [16], consolidating memory [17], modulating glial activation [18], and regulating neuroinflammation [19]. Conversely, the development of AD pathology may also adversely impact sleep processes, indicating a bidirectional relationship between sleep and AD [20, 21].

**Fig. 1:**
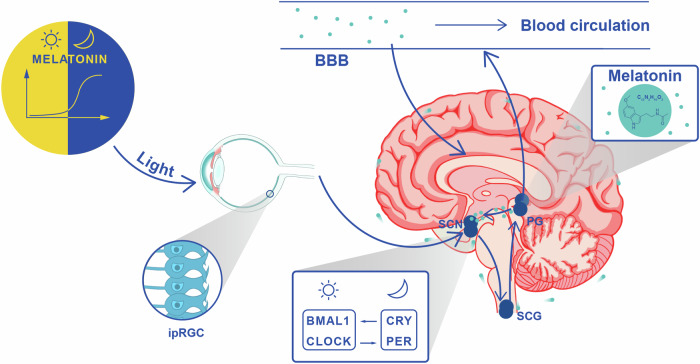
Light-driven regulation of melatonin synthesis. Light, particularly in the blue wavelength range (450-490 nm), a pivotal component of sunlight, activates intrinsically photosensitive retinal ganglion cells (ipRGC) situated in the retina of the eyes. These ipRGCs transform light stimuli into action potentials transmitted to the suprachiasmatic nucleus (SCN), positioned above the optic nerve crossing [243]. Individuals with Alzheimer’s disease (AD) often exhibit a significant loss of ipRGC. The SCN comprises of approximately 20,000 neurons in each hemisphere. The interplay of core clock genes, including *BMAL1*, *CLOCK*, *PER*, and *CRY*, not only governs 24-hour processes like gene transcription in SCN neurons but also orchestrates their regulatory impact on other brain regions [244, 245]. The intricate regulation of pineal melatonin synthesis and release relies on the signaling pathway originating from the SCN in the hypothalamus, extending to the pineal gland (PG) [246]. This process involves the intermediolateral cell column in the spinal cord and sympathetic input from the superior cervical ganglion (SCG), located near the base of the skull [247]. Once released into the bloodstream by the PG, melatonin exerts its influence on target cells through specific melatonin receptors, distributed in various central and peripheral tissues [248]. Notably, melatonin is released into both the blood and directly into the brain. Several factors can disrupt the body’s natural melatonin production, including nighttime light exposure, such as the use of light-emitting devices [249]. Advanced aging [250], medications (e.g., beta blockers) [251], and various medical conditions, including dementia, pain, cancer, and type 2 diabetes mellitus [252], can also impede melatonin production. Conditions that hinder the synchronization of endogenous melatonin production with the solar day, such as blindness [253], add an additional layer of complexity.

Melatonin, also known as N-acetyl-5-methoxytryptamine, is a key hormone in regulating sleep timing in humans [22] and may also play a role in the brain-health benefits associated with sleep. It is primarily produced in the pineal gland of the brain (Fig. 1) through a series of enzymatic reactions involving metabolites such as 5-hydroxytryptophan, serotonin, and N-acetylserotonin [23]. The hormone is mainly secreted into the bloodstream at night [22], affecting both peripheral organs and the central nervous system (CNS) due to its amphiphilic properties [24]. The pineal gland can also release melatonin directly into the brain ventricles, as shown by animal and human studies [25–27]. After being taken up by the liver from the bloodstream, melatonin undergoes enzymatic conversion to produce 6-hydroxymelatonin sulfate, which is then excreted in the urine [28].

Melatonin exerts its effects on target cells primarily through two G-protein-coupled receptors: MT1 and MT2 [29, 30]. These receptors are distributed throughout the body, including the brain [29, 30]. Activation of MT1 and MT2 receptors initiates various signaling pathways, including the inhibition of adenylate cyclase, which reduces the production of cyclic adenosine monophosphate, a crucial secondary messenger in many cellular processes [29, 30]. Beyond membrane-bound receptors, melatonin also interacts with intracellular proteins such as calmodulin and nuclear receptors, extending its biological activity [29, 30].

In addition to its role in sleep-wake regulation [22], melatonin demonstrates potential direct or indirect impacts on pathways implicated in AD pathology. As discussed in the following sections, this includes the potential to reduce the production of Aβ and the ability to counteract the effects of Aβ oligomers (Aβos), a highly pathogenic form of Aβ [31]. Additionally, melatonin may modulate the hyperphosphorylation of the microtubule-associated tau protein, a process leading to the formation of NFTs characteristic of AD [32]. Further contributing to its neuroprotective potential, melatonin reduces oxidative stress in the CNS. Its entraining effects on circadian processes in brain cells, aligning neural activity and modulating synaptic transmission with the natural day-night cycle, could also potentially mitigate the development of AD. The improved functioning of the BBB, enhanced clearance of metabolites and protein fragments implicated in AD from the brain - an astrocyte-dependent mechanism primarily occurring during sleep [14] - and enhanced insulin signaling, suggested to play a role in AD [33], present additional mechanisms by which melatonin may impact AD pathology.

Melatonin production declines with aging [34], possibly due to age-related calcification of the pineal gland [35], rather than increased clearance [34]. Notably, reduced melatonin levels and disrupted 24-hour melatonin rhythms are frequently observed in individuals with AD dementia [36–40]. Since there is an established link between disrupted melatonin rhythms and the risk of AD dementia [41, 42], investigating the potential therapeutic role of melatonin in addressing these issues is a promising area of research and intervention for AD. Consequently, our review delves into the diverse mechanisms through which melatonin could exert neuroprotective effects in the context of AD. Additionally, we examine current clinical trials and discuss existing gaps and opportunities that warrant further consideration.

Box 1 Sporadic Alzheimer’s disease In contrast to familial Alzheimer’s disease (AD), characterized by specific genetic mutations (such as those in genes encoding proteins involved in processes central to AD pathology, like presenilin and amyloid precursor protein) and typically manifesting with early onset [256], sporadic AD lacks a direct association with a clear genetic predisposition. The development of sporadic AD is predominantly attributed to an intricate interplay of genetic, environmental, and lifestyle factors [154, 257]. It is more widespread and tends to manifest later in life, constituting the majority of AD cases.

## Unlocking melatonin’s anti-amyloidogenic potential

AD is characterized by Aβ aggregation, which in turn is associated with synaptic loss, oxidative stress, and mitochondrial dysfunction in the brain [32, 43–47]. The neurodegenerative potential varies between different forms of Aβ peptides, for example, amyloid aggregates primarily comprise Aβ42, which has higher amyloidogenicity and lower solubility than Aβ40 [48, 49]. Amyloid aggregates form when the membrane-bound amyloid precursor protein (APP) undergoes a series of proteolytic cleavages orchestrated by β-secretase (beta-site APP-cleaving enzyme, BACE) and furthered by γ-secretase [50]. β-secretase-catalyzed cleavage produces soluble APPβ and C99, a membrane-bound fragment of APP [50]. Subsequently, γ-secretase acts on C99, releasing Aβ both outside and within the neuron [50]. In contrast, the non-amyloidogenic pathway is characterized by more active α-secretase-catalyzed cleavage (e.g., ADAM-10 and ADAM-17) of APP, releasing soluble APPα from neuronal membranes into the interstitial space of the brain. This initiates the production of C-terminal membrane-tethered α-secretase-derived fragment C83, which, upon additional enzymatic cleavage by γ-secretase, increases the concentration of p3 [50]. A third pathway involving cleavage of APP by β- and α-secretase independently of γ-secretase is also non-amyloidogenic and results in the secretion of 14-16 amino acid-long Aβ [51].

While typically not considered in conventional models of amyloid plaque formation, melatonin intersects with several pathways. For example, melatonin has been found to stimulate the α-secretase cleavage of βAPP in cultured neuronal and non-neuronal cells [52] via upregulation of the nonamyloidogenic ADAM10 and ADAM17 proteases. In turn, presence of α-secretase inhibitors in these cell lines abrogates the α-secretase-dependent activation of the non-amyloidogenic pathway by melatonin [52]. In studies using SH-SY5Y cells, recognized as a model for studying neurodegenerative processes [53], melatonin exhibits an inhibitory effect on the expression of amyloidogenic β-secretases [54]. The increased activity of α-secretases and concurrent reduction in the activity of β-secretase may partially explain why melatonin treatment results in diminished Aβ levels in the brains of both sporadic and transgenic animal models of AD [55–57]. It is also worth noting that Aβ peptides have been shown to significantly decrease the production of melatonin by the pineal gland [58], underscoring the bi-directional nature of these relationships.

Melatonin appears to modulate the activity of enzymes engaged in the modification of membrane-bound APP in neurons, including glycogen synthase kinase-3β (GSK3β). GSK3β, a serine-threonine kinase prominently expressed in the brain, augments the activity of β-secretase while concurrently diminishing the activity of α-secretases, thereby amplifying intra- and extracellular levels of Aβ [59, 60]. In a murine model of AD, melatonin diminished GSK3β activity [61], an effect potentially reliant on melatonin receptor MT1, as implied by findings in SH-SY5Y cells [62]. Using SH-SY5Y cells exposed to high glucose concentrations to induce hyperglycemia, disruptions in glucose sensing leads to activation of the phosphorylated protein kinase B (pAkt)/GSK-3β signaling pathway, and increased expression of BACE and Aβ42, an effect that can be reversed via pretreatment with melatonin [62]. Moreover, melatonin may impact amyloid clearance via its impact on insulin-degrading enzyme (IDE), a protease pivotal for catalyzing extracellular and intracellular Aβ degradation [63]. IDE exhibits reduced abundance concomitant with elevated CNS activity of GSK3β in diabetic murine models [64]. Significantly, a compound denoted as melatonin-trientine, covalently synthesized with melatonin and the metal ion chelator trientine, demonstrated the capacity to elevate the expression of IDE in a murine AD model, and decreased Aβ deposition and neuronal degeneration in the brains of the APP/Presenilin 1 mice [65].

Another contributing mechanism to melatonin’s anti-amyloidogenic effects may involve the enhanced clearance of Aβ from the brain. Astrocytes, positioned at the interface between the brain parenchyma and the perivascular space, which includes capillaries, larger arteries, and veins [66], play a role in the removal of Aβ from the brain via mechanisms that include enzymatic degradation of Aβ and the upregulation of efflux transporters for Aβ at the BBB [67]. Studies conducted with mouse neuroblastoma cells (Neuro-2a cells) suggest that melatonin has the potential to augment the expression of Transcription Factor EB [68]. This transcription factor acts as a master regulator of lysosome biogenesis, thereby promoting autophagosome-lysosome clearance of Aβ by astrocytes [69].

Melatonin may exert an additional impact on Aβ clearance by upregulating the expression of the low-density lipoprotein receptor-related protein 1 (LRP1) [70]. This transporter may play a crucial role in both the uptake of Aβ into astrocytes and the efflux of Aβ from the brain at the BBB [71]. Apolipoprotein E (ApoE), a protein interacting with cell surface receptors to facilitate the uptake of lipoproteins [72], is postulated to hinder the clearance of soluble Aβ from the brain by competing with soluble Aβ for LRP1–dependent cellular uptake into astrocytes [73]. Consequently, this competition heightens the likelihood of Aβ aggregation in the brain, ultimately leading to plaque formation. Notably, research involving astrocytes cultured from a transgenic AD mouse model overexpressing *apoE* demonstrated that melatonin reversed the Aβ aggregation-promoting activity of this protein [74]. The observation that patients with AD carrying two alleles of the *APOE4* risk variant have approximately half the cerebrospinal fluid (CSF) melatonin levels compared to those with only one allele of this variant [75] could suggests that *APOE4/4* carriers might particularly benefit from supplementing with exogenous melatonin.

**Fig. 2:**
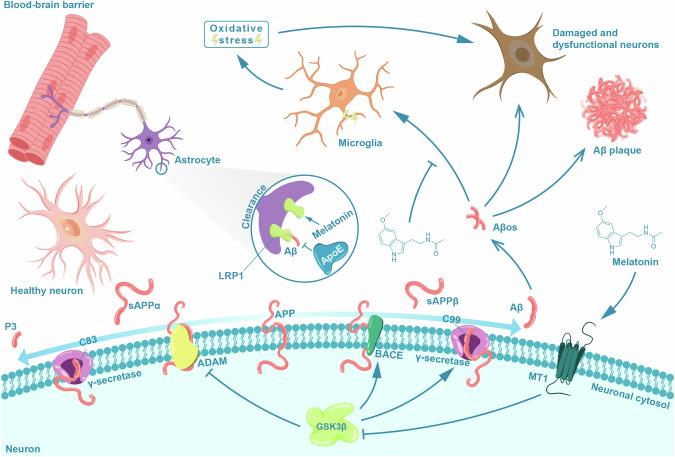
Melatonin modulation of amyloid beta production and clearance in Alzheimer’s disease. In the pro-amyloidogenic pathway, membrane-bound amyloid precursor protein (APP) undergoes proteolytic cleavages orchestrated by β-secretase (BACE) and γ-secretase, leading to increased intraneuronal and brain interstitial fluid amyloid-beta (Aβ) concentrations. If enzymatically cleaved first by alpha (ADAM) and then γ-secretase, no Aβ is produced. If cleaved concertedly by β- and α-secretases, short 14-16 amino acid-long Aβ fragments form but they are highly hydrophilic and not part of any amyloidogenic cascade. Through self-assembly, soluble Aβ oligomers (Aβos) are formed, known to disrupt neuronal signaling through excitotoxicity and induce oxidative stress (e.g., through microglial activation), thereby exacerbating the process of neurodegeneration. Aβos can further assemble to build fibrils, which represent a major component of Aβ plaques [254, 255]. As reviewed in Section *Unlocking Melatonin’s Anti-Amyloidogenic Potential* and Section *Melatonin’s Neuroprotective Mechanisms against Aβ toxicity*, melatonin redirects APP toward the non-amyloidogenic pathway, potentially achieved by suppressing glycogen synthase kinase-3β (GSK3β) activity. This is achieved through various mechanisms, e.g., the activation of the membrane-bound melatonin receptor 1. GSK3β is a kinase known to promote the pro-amyloidogenic pathway. Consequently, melatonin reduces Aβ burden in the brain. Melatonin may also increase the clearance of Aβ. For example, treatment with melatonin has been shown to upregulate transporters such as the low-density lipoprotein receptor-related protein 1 (LRP1), which plays a decisive role in the uptake of soluble Aβ proteins from the brain interstitial fluid into astrocytes. In animal models that overexpress apolipoprotein E (ApoE), a variant recognized for competing with Aβ for astrocytic uptake, melatonin has been shown to alleviate Aβ burden, most likely achieved through the upregulation of LRP1. Melatonin has also been demonstrated to mitigate processes involved in Aβos’ adverse effects on brain cells, such as excitotoxicity and oxidative stress.

In addition to its impact on astrocytic clearance, melatonin may mitigate the burden of Aβ in the brain by promoting lymphatic clearance. In the transgenic AD mouse model Tg2576, marked by mutant APP overexpression in the brain, melatonin treatment exhibited a trend of elevated Aβ levels in cervical and axillary lymph nodes, concomitant with a decreasing trend in brain Aβ [76]. Figure 2 provides a summary of the mechanisms that may explain the anti-amyloidogenic effects of melatonin.

## Melatonin’s neuroprotective mechanisms against Aβ toxicity

According to the current version of the Aβ cascade hypothesis, diffusible oligomeric forms of Aβ (Aβ oligomers, Aβos) represent the most pathogenic and toxic form of Aβ [31]. Aβos, upon binding to cell-surface receptors on neurons, disrupt signal transduction, leading to a sustained interruption of intracellular Ca^2+^ signaling through increased N-methyl-D-aspartate (NMDA) receptor-dependent Ca^2+^ influx [77]. Ca^2+^-dependent hyperexcitability can contribute to cellular damage and cell death through various mechanisms, including mitochondrial dysfunction and apoptosis [78]. In a rat model, melatonin demonstrated inhibition of NMDA receptor activation and subsequent NMDA receptor-mediated Ca^2+^ entry into striatal neurons, suggesting a potential protective role [79]. However, melatonin has also been shown to upregulate NMDA receptor subunits 2A and 2B in a dose-dependent manner in the rat hippocampus [80].

In addition to excitotoxicity, Aβos can inflict injury to brain cells through oxidative stress. Specifically, Aβos possess the ability to bind to metal ions, thereby promoting the redox activity of these metals [81–83]. Moreover, oligomeric forms of Aβ can increase oxidative stress in neurons [84, 85]. For example, mitochondria isolated from the rat whole brain, when exposed to Aβos, produce more reactive oxygen species (ROS) [86]. Another mechanism through which Aβos contribute to oxidative stress involves the activation of toll-like receptor 4 (TLR4) on microglia [87], the brain’s resident innate immune cells. TLR4 activation triggers various pro-inflammatory processes in microglia, including an augmented production of ROS [88].

As a consequence of heightened oxidative stress induced by Aβos, the transient receptor potential melastatin 2, a Ca^2+^-permeable channel expressed on brain cells, opens [89]. If prolonged, this can elevate the risk of neuronal dysfunction and damage due to excessive Ca^2+^ influx into neurons [78]. Furthermore, escalated oxidative stress can activate GSK3β [90], contributing to elevated Aβ levels [60] (see Section *Unlocking Melatonin’s Anti-Amyloidogenic Potential*) and hyperphosphorylation of the microtubule-associated protein tau [91, 92] (see *Melatonin’s Potential to Counteract Tau Pathology*). Melatonin, known for its robust scavenging of ROS and reactive nitrogen species (RNS) [93], can effectively penetrate brain cells owing to its amphiphilic biochemical nature [94]. The reduction in oxidative stress, therefore, serves as an additional mechanism by which melatonin safeguards brain cells against the toxic effects of Aβos [95].

In addition to accumulation of amyloid and tau pathology, AD involves an immune response. Accumulation of pathology including Aβ triggers activation of microglia in the brain [96]. Activated M1 (or pro-inflammatory) microglia are part of an inflammatory cascade that includes upregulation of pro-inflammatory genes, release of pro-inflammatory cytokines, and generation of ROS, which may, under prolonged conditions, contribute to the neuroinflammation observed in AD brain [97]. Additionally, M1 microglia are often associated with reduced phagocytic activity, which may also make microglia less effective at clearing Aβos and plaques [98]. Conversely, the M2 phenotype is generally considered anti-inflammatory and has been associated with enhanced phagocytic activity, potentially reducing the risk of AD and its progression [99]. In this context, the triggering receptor expressed on myeloid cells 2 (TREM2) may play a major role in promoting the M2 microglia phenotype [99]. Consequently, a lack of function of TREM2 has previously been linked to an increased risk of AD [100]. Notably, as suggested in an animal model of neuroinflammation induced by ischemic stroke damage known to increase the prevalence of M1 microglia in the brain, pre-treatment with melatonin upregulated the expression of TREM2 while downregulating that of proinflammatory genes in microglia (e.g., inducible nitric oxide synthase) [101], suggesting that melatonin may polarize microglia towards its M2 phenotype.

The evidence outlined in the Sections *Unlocking Melatonin’s Anti-Amyloidogenic Potential* and *Melatonin’s Neuroprotective Mechanisms against Aβ toxicity* indicates that melatonin exhibits anti-amyloidogenic properties and mitigates the neurotoxic effects of Aβos, thereby potentially reducing the risk of developing AD or slowing its progression. It is imperative to emphasize that these findings predominantly originate from animal studies and experiments conducted with cell lines. As of the present moment, there are no published studies that have investigated the impact of melatonin on Aβ burden specifically in humans.

## Melatonin’s potential to counteract tau pathology

The microtubule-stabilizing tau protein assumes a critical role in the assembly and stabilization of microtubules, integral components of the neuronal cytoskeleton [102]. Under normal physiological conditions, tau harbors merely 2–3 phosphate groups. However, in tauopathies such as AD, as well as in hibernation, tau undergoes hyperphosphorylation catalyzed by various protein kinases, including but not limited to glycogen GSK3β, cyclin-dependent kinase 5 (CDK5), and protein kinase A (PKA) [103–105], accumulating an excess of 7-10 phosphate groups [106]. As tau becomes hyperphosphorylated, its capacity to support microtubule assembly and stability diminishes, resulting in increased levels of unbound tau that may aggregate into tangles (if not secreted or degraded in the lysosome) [107]. This disruption assumes a pivotal role in the neurodegenerative processes observed in AD and other tauopathies [108, 109]. Hyperphosphorylated tau and tangle development are more closely associated with synaptic dysfunction and cognitive decline (including conversion to mild cognitive impairment and AD dementia) compared to Aβ alone [110–112].

As evidenced by animal studies and cell line experiments, melatonin exhibits promise in mitigating tau hyperphosphorylation. For instance, intraperitoneal pretreatment with melatonin prevented isoproterenol (a beta-receptor agonist)-induced tau hyperphosphorylation in rats [113]. Another study, involving the pharmacological blocking of endogenous melatonin production in rats, observed an increase in tau hyperphosphorylation. This effect was reversed when melatonin-deficient animals were supplemented with melatonin [114]. In a transgenic mouse model exhibiting age-associated tau pathology, melatonin treatment efficiently decreased the hyperphosphorylation of tau [115]. Additionally, melatonin significantly reduced the number of NFTs and attenuated neuronal loss in the cortex and hippocampus [115]. When SH-SY5Y cells were exposed to mercury, inducing a two-fold increase in tau hyperphosphorylation over a 9-hour period, pre-treatment with melatonin for 12 hours effectively reduced tau hyperphosphorylation due to this transition metal [116]. Lastly, among rats, the administration of wortmannin (a fungal metabolite) which induces hyperphosphorylation of tau in hippocampal pyramidal neurons, can be partly attenuated by preinjection of melatonin [117, 118], underscoring melatonin’s potential therapeutic role.

**Fig. 3:**
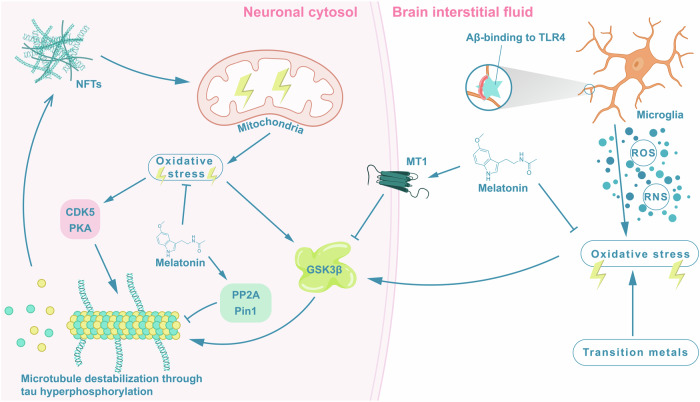
Melatonin’s role in mitigating tau hyperphosphorylation and neurofibrillary tangles formation in Alzheimer’s disease. Tau, a microtubule-associated protein crucial for the structural integrity of neurons, undergoes hyperphosphorylation in Alzheimer’s disease (AD), leading to the accumulation of phosphorylated tau and the formation of neurofibrillary tangles (NFTs). This hyperphosphorylation compromises tau’s ability to support microtubule assembly, contributing to the development of NFTs in neurons. Furthermore, NFTs and hyperphosphorylated tau can induce oxidative stress in neurons, primarily through mitochondrial dysfunction. Oxidative stress may also activate kinases associated with tau hyperphosphorylation, including glycogen synthase kinase-3β (GSK3β), cyclin-dependent kinase 5 (CDK5), and protein kinase A (PKA). Increased oxidative stress acting on neurons can also result from amyloid β (Aβ)-driven activation of microglia through toll-like receptor 4 (TLR4) or interactions with transition metals in the brain interstitial fluid, leading to an abundance of reactive oxygen species (ROS) and reactive nitrogen species (RNS). As elucidated in Section *Melatonin’s Potential to Counteract Tau Pathology*, melatonin exhibits promising potential in mitigating tau hyperphosphorylation, as demonstrated in both animal models of AD and cell line experiments. Through its robust antioxidative potential, melatonin may hinder the oxidative stress-induced activation of protein kinases involved in tau hyperphosphorylation. Additionally, the hormone, via activation of its melatonin receptor 1, reduces the activity of GSK3β, showcasing another anti-tauogenic effect. Melatonin further engages in the activation of enzymes that counteract tau hyperphosphorylation. For instance, it upregulates the activity of phosphatase 2 A (PP2A), recognized for its role in dephosphorylating tau, and elevates levels of Peptidyl-Prolyl cis-trans Isomerase NIMA-Interacting 1 (Pin1), crucial for restoring tau function.

Various mechanisms may underlie the inhibitory effect of melatonin on tau hyperphosphorylation, as depicted in Fig. 3. In neuroblastoma SH-SY5Y cells exposed to hyperglycemia, a condition known to increase GSK3β activity [119], melatonin exerted an inhibitory effect on the GSK3β signaling pathway, likely through the activation of its melatonin receptor [62]. GSK3β serves as a key kinase in the hyperphosphorylation of the microtubule-associated protein tau, thereby contributing to neuronal dysfunction and degeneration [91, 92]. Stimulation of GSK3β activity can occur through exposure to oxidative stress [90]. Within neurons, oxidative stress may stem from mitochondrial dysfunction induced by the intracellular accumulation of hyperphosphorylated tau and Aβ [84, 120]. Furthermore, interactions of brain interstitial Aβ with microglia [87, 88] and exposure to transition metals [81–83] also contribute to oxidative stress in neurons. Thus, melatonin’s dual capacity to act both as a hormone and antioxidant may counter GSK3β-driven tau hyperphosphorylation in neurons. Notably, melatonin also diminished the activity of PKA in a rat model [121], a known trigger for tau hyperphosphorylation by GSK3β [122, 123]. Additionally, melatonin reduced the activities of other kinases involved in tau hyperphosphorylation, such as CDK5, through the upregulation of micro-RNAs [115].

Melatonin’s impact extends to various enzymes involved in mitigating tau hyperphosphorylation. For instance, a study in rats suggests that melatonin may counteract tau pathology by upregulating the activity of phosphatase 2A [114], an enzyme recognized for its role in dephosphorylating microtubule-associated protein tau [124]. Additionally, in SH-SY5Y cells, melatonin increased levels of Peptidyl-Prolyl cis-trans Isomerase NIMA-Interacting 1 (Pin1) [125], which has been shown to mitigate tau phosphorylation and aggregation [126]. The absence of Pin1 has been associated with age-dependent tau hyperphosphorylation and neurodegeneration [126].

Although the described findings predominantly originate from in cell line studies and animal experiments, a recent clinical study involving around 80 patients with mild cognitive impairment suggests that melatonin supplementation may also exhibit anti-tauogenic potential in humans. In particular, the study revealed that individuals who received a daily evening dose of 0.15 mg of melatonin per kg of body weight over six consecutive months exhibited CSF levels of tau proteins compared to those treated with a placebo [127].

## Crossroads between melatonin and insulin

Insulin, a hormone primarily synthesized by pancreatic β-cells, has demonstrated notable efficacy in counteracting processes implicated in AD pathology upon reaching the brain [33]. Augmentation of brain insulin signaling enhanced cognitive functions in both cognitively healthy individuals and those with AD [128, 129]. Furthermore, insulin plays a crucial role in mitigating synaptic vulnerability to Aβos in hippocampal neurons [130, 131]. Supporting this, studies indicate that treatment with insulin or insulin-sensitizing drugs effectively reduced tau hyperphosphorylation in neurons [132, 133]. Conversely, a deficiency of brain insulin, as observed in transgenic mice, was associated with an increase in tau hyperphosphorylation, likely due to increased GSK3β activity [134].

Significantly, melatonin supplementation has demonstrated the capacity to alleviate oxidative stress and impede the accumulation of Aβ, as well as tau hyperphosphorylation, in the hippocampus of aged rats exhibiting brain insulin resistance induced by a high-fat diet [135]. The discovery that melatonin enhances the survival and function of insulin-producing β-cells [136] adds more support to the idea that melatonin plays a role in regulating insulin signaling. Particularly noteworthy is the discovery that melatonin signaling in β-cells led to a decrease in the activation of the stress response c-Jun N-terminal kinase [136]. This pathway is known to play a pivotal role in inducing brain insulin resistance in AD [131]. Further supporting melatonin’s potential impact on brain insulin, a rat study demonstrated that melatonin, upon binding to its membrane-bound receptors, can initiate intracellular pathways in the brain typically activated by insulin binding to the insulin receptor (e.g., AKT serine phosphorylation) [137].

As shown by animal studies, insulin may also play a crucial role in promoting melatonin synthesis in the pineal gland. Insulin can enhance norepinephrine-mediated melatonin production in the pineal gland of rats [138]. Additionally, insulin facilitates the transport of tryptophan into the brain, which is an essential precursor for melatonin synthesis [139].

Collectively, the evidence presented suggests that melatonin’s ability to mitigate AD may involve the modulation of whole-body insulin pathways, including the brain. However, it is essential to note that sustained elevation in plasma glucose concentrations due to reduced pancreatic insulin release from chronic melatonin treatment, especially when extending beyond the sleeping period [140], may compromise melatonin’s potential to ameliorate AD pathology [141, 142].

## Oxidative stress - implications and mitigation with melatonin

Oxidative stress is characterized by an imbalance between the production of reactive species (e.g., ROS and RNS) and the body’s antioxidant defenses [143]. In the brain, prolonged oxidative stress initiates various pathological processes that play a pivotal role in AD pathology. Neuronal response to oxidative stress may activate GSKβ3, leading to the redirection of membrane-bound APP into the pro-amyloidogenic pathway (see Section *Unlocking Melatonin’s Anti-Amyloidogenic Potential* and Fig. 2). This results in an increase in intra- and extracellular Aβ. GSKβ3 also catalyzes the hyperphosphorylation of tau (see Section *Melatonin’s Potential to Counteract Tau Pathology* and Fig. 3), elevating the risk of NFTs. Prolonged oxidative stress can also induce apoptosis in neurons [144] and trigger neuroinflammation [145], both common characteristics in the brains of patients with AD [146, 147]. Consequently, countering prolonged oxidative stress emerges as a promising strategy to mitigate multiple processes associated with AD pathology.

In the context of CNS oxidative stress, melatonin emerges as a surprisingly prominent player. Distinguished as a potent non-enzymatic antioxidant and free radical scavenger, melatonin possesses the unique ability to neutralize free radicals independently of receptors, owing to its distinctive chemical structure [93]. This intrinsic function enables melatonin to directly detoxify ROS and RNS [93]. Leveraging its amphiphilic biochemical property [94], melatonin not only traverses the BBB but also gains access to brain cells and brain cell organelles, including mitochondria. This dual capability positions melatonin as a robust non-enzymatic antioxidant, functioning both intra- and extracellularly [93].

Furthermore, melatonin indirectly combats free radicals by chelating metal ions in the brain [148]. It also modulates the activity of pro-oxidative enzymes like nitric oxide synthase, while enhancing the activities of antioxidant enzymes, including glutathione peroxidase and superoxide dismutase in neurons [149–152]. Noteworthy findings from a mouse model of traumatic brain injury, a condition associated with increased oxidative stress in affected brain areas [153] and considered a risk factor for AD [154], underscore that melatonin treatment enhances the expression of nuclear factor erythroid 2-related factor 2 (Nrf2) [155]^142^. Nrf2, a redox-sensitive transcription factor, is pivotal in transcribing numerous antioxidant genes. The downregulation of Nrf2 is implicated in fostering oxidative stress in AD pathology [156].

Neuronal apoptosis also triggers oxidative stress [157]. Neuronal cell death activates microglia [158], leading to the production of more reactive species and the release of pro-inflammatory cytokines [159]. Melatonin has been shown to inhibit the expression of key signals involved in initiating apoptosis in rodent models of AD and various brain cell types. This includes Bax, a pro-apoptotic protein, and caspase-3, a key enzyme in apoptosis [160–164].

Notably, a study in Aβ plaque-bearing Tg2576 mice, initiated at 14 months of age, showed no significant impact on relieving oxidative damage, even with elevated plasma melatonin levels [165]. However, it was found effective in countering oxidative stress in a transgenic mouse model of amyloidosis when administered from four months of age [166]. These findings suggest that melatonin’s antioxidative effects may be more therapeutically relevant early during the disease.

## Melatonin - A circadian shield

Virtually all cells within the body exhibit a robust circadian pattern, orchestrating the timing of various cellular processes throughout the day [167]. See Box 2 for a definition of circadian rhythms. Brain cells, including neurons, microglia, and astrocytes, also adhere to circadian rhythms [168–170]. To ensure synchronization among brain cell clocks, various mechanisms, including the release of melatonin by the pineal gland [171], come into play.

Among individuals with AD, pathways governing melatonin regulation and production may be affected. For instance, a reduction in the number of intrinsic photosensitive retinal ganglion cells has been observed in AD patients [172]. Additionally, there is evidence of structural and functional loss of neurons in the suprachiasmatic nucleus (SCN) in this disease [40, 173]. The SCN, located above the optic nerve crossing, plays a pivotal role in regulating the timing of sleep and wakefulness [174]. Neurodegeneration affecting the pineal gland could be an additional factor contributing to the observed lower melatonin levels in AD patients compared to age-matched non-demented controls [175].

Disrupted circadian rhythms in brain cells, stemming from impaired functioning of the retina-SCN-pineal axis, may contribute to both neurobehavioral symptoms and neurodegenerative processes commonly observed in AD. For instance, many AD patients display behavioral symptoms such as rest-activity rhythm fragmentation [176], sundowning (characterized by recurring confusion or agitation in the late afternoon or early evening) [177], and disturbances in sleep and wake maintenance [178]. Additionally, circadian disruption might promote neurodegenerative processes implicated in AD development, as indicated by recent animal studies. For example, the deletion of the core clock gene *Bmal1* in the mouse brain led to astrocyte activation - an established marker of brain and neuronal injury - as well as synaptic degeneration [179]. Mass spectrometry analysis revealed a threefold increase in markers of neuronal membrane lipid peroxidation in *Bmal1* knockout mice, reflecting heightened neuronal oxidative damage [179]. The increased oxidative stress in neurons was partially attributed to the reduced expression of redox defense genes [179]. In a separate study [180] using a mouse model of amyloidosis and disrupted circadian clock function through an inducible whole-organism deletion of the core clock gene *Bmal1*, these animals accumulated a higher hippocampal Aβ plaque burden. Furthermore, the deletion of *Bmal1* in the brain induced *apoE* expression (inhibiting, e.g., astrocytic clearance of Aβ [73]), potentially elucidating how circadian disruption may increase Aβ burden in the brain.

While brain interstitial fluid tau concentrations are reported to be nearly twice as high during the active period of the rest-activity cycle in humans [8], it is noteworthy that, to the best of our knowledge, no study has investigated whether the deletion of core clock genes, simulating circadian disruption, leads to an increase in tau hyperphosphorylation. Despite this gap in knowledge, a plausible hypothesis could be formulated that either treatment with exogenous melatonin or interventions aimed at restoring endogenous melatonin secretion might offer potential in mitigating neurodegenerative processes induced by circadian disruption.

Box 2 Circadian rhythms Circadian rhythm, derived from the Latin term *circa diem*, meaning “about a day,” pertains to roughly 24-hour cycles driven by recurring environmental cues such as light and food intake. These cycles synchronize and are inherent to most physiological processes, constituting endogenous rhythms. Melatonin, secreted by the pineal gland, plays a crucial role in regulating circadian rhythms. Its release is influenced by the light-dark cycle, contributing to the synchronization of various biological activities with the day-night cycle.

## Melatonin’s role in safeguarding the BBB integrity and functionality

The BBB is a highly intricate structure composed of endothelial cells, pericytes, the capillary basement membrane, and astrocyte end-feet [181]. Its primary role is to serve as a crucial defense mechanism, preventing harmful substances from entering the brain while facilitating the transport of essential nutrients to brain tissue [181]. However, factors such as advanced aging, peripheral inflammation, and cerebral amyloid angiopathy can compromise the integrity of the BBB [182, 183]. A dysfunctional BBB can accelerate neuronal degeneration and cognitive decline, creating conditions that favor the accumulation of Aβ and the infiltration of neuroinflammatory agents and other molecules into the brain [184]. This disruption is hypothesized to contribute to the onset of various neurological disorders, including AD [185].

Melatonin demonstrates effectiveness in mitigating blood-brain barrier (BBB) damage through several mechanisms. Studies show that melatonin activates receptors to boost P-glycoprotein transporter activity, crucial for BBB function, particularly in methamphetamine-induced toxicity in rat brain endothelial cells [186]. Additionally, melatonin protects the BBB from methamphetamine damage by inhibiting nicotinamide adenine dinucleotide phosphate oxidase 2 via its receptors in these cells [187]. Melatonin’s protective effects on the BBB have also been confirmed in various experimental models. For instance, in a neonatal rat model, melatonin effectively reduced BBB damage caused by excitotoxicity [188]. Similarly, in young mice subjected to transient focal cerebral ischemia, melatonin demonstrated significant protective effects on the BBB [189, 190].

Melatonin’s potential to strengthen the BBB appears to involve the upregulation of tight junction proteins, including claudin-5, zonula occludens-1, and occluding [191], which help shield the brain from harmful substances in the bloodstream [192]. Additionally, melatonin may help maintain BBB integrity and function by interfering with angiotensin-converting enzyme 2, which acts as a receptor for viral entry. Daily injections of melatonin and melatonergic compounds have been shown to significantly reduce angiotensin-converting enzyme 2-dependent viral entry of severe acute respiratory syndrome coronavirus 2 into the brain [193]. This reduction is significant because this virus has been shown to exacerbate Aβ neurotoxicity and oxidative stress in patients with AD [194]. Melatonin also regulates matrix metalloproteinases (MMPs), enzymes known to compromise BBB integrity [195]. In a human gastric adenocarcinoma cell line, melatonin reduced MMP activity [196]. Furthermore, in aged mice treated with lipopolysaccharide to increase BBB permeability, melatonin-activated AMP-activated protein kinase in endothelial cells of the BBB [197], a kinase essential for maintaining BBB integrity [198].

In summary, these findings highlight melatonin’s role in protecting the BBB and mitigating damage across various experimental models. They underscore its potential as a therapeutic agent for maintaining BBB integrity and preventing neurological deterioration.

## Melatonin and functioning of the glymphatic system

The role of sleep in brain waste clearance has been studied for over a decade, with recent studies suggesting a potential role for melatonin in glymphatic function. Originating in the subarachnoid space, CSF traverses periarterial spaces, mixes with interstitial fluid in the brain parenchyma, and ultimately exits via perivenous spaces [199]. While the first studies showing this were in mice [14], human studies have corroborated these findings [200], although its importance in relationship to traditional clearance pathways across the BBB and subarachnoidal granulations has been challenging to quantify.

Functioning as a clearance mechanism for the brain, the glymphatic system assists in removing potentially harmful waste products, including soluble Aβ, which accumulate in the interstitial fluid of the brain during the day [2, 21, 201, 202]. At the core of this process are aquaporin-4-expressing astrocytes enveloping perivascular spaces [203]. Animal studies have emphasized the efficiency of the glymphatic system in purging waste from the brain, with peak effectiveness during sleep [14], particularly at night [204], and reduced efficacy observed in animal models of hypertension [205]. However, the importance of sleep for the glymphatic system has not been confirmed by all investigations [206].

Melatonin, implicated in signaling the biological night to various cells by inducing hypothermia and promoting sleepiness [207, 208], may lower the release of the wake-promoting neuropeptide orexin, according to animal research [209]. Elevated orexin levels during sleep have been linked to increased Aβ pathology in mice brains [2, 210], possibly due to reduced glymphatic system efficacy [211]. Evidence in hypertensive patients suggests that melatonin can lower nocturnal blood pressure [212, 213]. These effects, in conjunction with potential sleep-enhancing benefits observed in patients with dementia treated with melatonin [214], provide a compelling rationale for hypothesizing that supplementing melatonin near bedtime may augment the glymphatic system’s clearance function. Supporting this hypothesis, a chronic unpredictable mild stress mouse model demonstrated that melatonin effectively restored aquaporin-4 polarization and rectified the compromised glymphatic system observed in this model [215]. Nonetheless, clinical trials involving both healthy individuals and those with mild cognitive impairment or AD are warranted to investigate whether melatonin can enhance the glymphatic system’s efficacy in reducing brain concentrations of Aβ and tau.

## From animal models to clinical trials: the promise of melatonin in cognitive health

In animal models of AD, melatonin has shown promise in improving spatial memory and mitigating cognitive impairments. For example, in a sporadic AD mouse model induced by D-galactose and aluminum chloride, melatonin significantly enhanced short-term spatial memory. This improvement was linked to increased hippocampal expression of the memory-associated genes cAMP-responsive element-binding protein and brain-derived neurotrophic factor [216].

A systematic review and meta-analysis of nine studies involving 294 animals demonstrated that melatonin significantly improved learning abilities and corrected memory deficits in AD models [217]. This was evidenced by reduced escape latency, increased dwell time in the target quadrant, and more crossings over the platform location in the Morris Water Maze test. Melatonin was most effective in enhancing learning ability in senescence-related and metabolic AD models and in correcting memory deficits in toxin-induced AD models.

Studies on developing rats have also shown that melatonin can alleviate spatial learning and memory impairments by suppressing isoflurane-induced endoplasmic reticulum stress through the Sirtuin 1/Mitofusin 2/Protein kinase RNA-like endoplasmic reticulum kinase signaling pathway [218].

The positive effects of melatonin on cognition have also been documented in various human cohorts, including elderly subjects and patients with AD. A meta-analysis of 22 randomized controlled trials highlighted that patients with AD who received more than 12 weeks of melatonin treatment showed improvements in Mini-Mental State Examination scores, particularly in those with mild AD [219]. Additionally, a cross-sectional study of 1,105 community-dwelling elderly individuals found that higher physiological melatonin levels were correlated with a lower prevalence of depressed mood and cognitive impairment, independent of depressive symptoms [220].

A double-blind, placebo-controlled pilot study involving 26 healthy elderly subjects revealed that participants who received 1 mg of melatonin nightly for four weeks showed improved performance on the California Verbal Learning Test-interference subtest compared to those who received a placebo [221]. Further research demonstrated that melatonin enhances recognition memory accuracy for objects encoded under stress in healthy young men, indicating its role in central nervous system processing during stress and its potential to modulate memory consolidation [222].

A study involving individuals undergoing hemodialysis found that after six weeks of taking 3 mg of melatonin before bedtime, cognitive function significantly improved, with the Montreal Cognitive Assessment score increasing from 21.19 to 24.27 in the intervention group, compared to 22.15 in the control group [223]. Moreover, a study of 52 cognitively healthy adults, averaging 70 years of age, revealed a positive correlation between greater melatonin levels 6 hours before habitual bedtime and hippocampal volume - a region susceptible to AD pathology and integral to memory function [224, 225]. Finally, a network meta-analysis incorporating data from 50 randomized placebo-controlled trials involving approximately 20,000 AD patients demonstrated that the administration of melatonin (≤3 mg/day) over a period of 6 to 12 months was associated with improved cognitive function [226].

The evidence from both animal and human studies suggests that melatonin has significant potential as a therapeutic agent for improving cognitive function and mitigating cognitive impairments associated with AD.

## Unlocking melatonin’s potential: evidence from observational studies and trials

The use of melatonin in older adults is increasing over time. In the US, the prevalence of melatonin use for sleep disorders among individuals 65 or older increased from 0.6% in 1999 to 2.1% in 2018, with a similar rise in use in UK. Melatonin appears to be a safe medication, particularly when compared to other sleep aids, but it remains understudied in older adults who show higher absorption compared to younger adults [227].

Observational studies in humans, while limited in number, provide intriguing evidence supporting a role for melatonin in the context of AD. In particular, studies showing diminished 24-hour melatonin levels or disrupted melatonin rhythm - where the primary endogenous melatonin release fails to synchronize with the dark phase of the daily light-dark cycle - have been correlated with an elevated risk of AD. Notably, a study involving approximately 276,000 participants from the UK Biobank cohort revealed that permanent night shift work, a condition acknowledged to reduce 24-hour melatonin levels [41], was associated with a 1.5-fold higher risk of developing AD during a median follow-up of 9 years [42]. Furthermore, the observation that visual loss is associated with an increased longitudinal risk of AD diagnosis, with a greater risk observed when visual loss occurs earlier in the lifespan [228], accentuates the potential significance of aligning the endogenous melatonin rhythm with the light-dark cycle for optimal brain health in humans.

Clinical investigations have shown that melatonin concentrations in the CSF of patients with AD are several-fold lower than those in age-matched control subjects without AD [75, 229]. Furthermore, lower CSF melatonin levels in AD patients are correlated with greater disease severity [229]. However, it remains unclear whether this reduction is due to decreased release from the pineal gland, increased breakdown of the hormone, or a combination of both factors.

Insights from a clinical trial involving nearly 200 nursing home residents in the Netherlands (mean age: 86; 87% with dementia) highlight the potential benefits of a daily evening regimen of melatonin (2.5 mg) combined with daytime bright light exposure over an average period of 15 months [214]. This regimen was associated with a reduction in agitated behavior - a common symptom in individuals with AD [230] - and improvements in sleep. However, some studies have failed to find an effect of melatonin on sleep in patients with AD [231, 232]. This discrepancy may be due to AD-related injury at later disease stages, emphasizing the need for clinical trials initiated prior to dementia onset. Another possibility is that patients may respond less to melatonin, particularly if they carry genetic polymorphisms associated with reduced expression of melatonin receptors [233].

As noted above, the majority of studies examining the impact of melatonin on AD-related mechanisms have been carried out in cell lines and animal models. Given the availability of biomarkers for AD that can be used to measure brain pathology in vivo (including neuroimaging with positron emission tomography, CSF, and plasma biomarker analysis), studies are needed in older adults which measure AD biomarkers in the context of melatonin use. Given its high potential for impacting AD-relevant pathologies with low risk for adverse events, additional studies of melatonin in the context of AD appear warranted.

## Conclusion

AD stands as a substantial global health and economic challenge [234, 235], emphasizing the pressing need for effective therapeutic interventions and preventive strategies. Melatonin, recognized for its dual role as an antioxidant and hormone, emerges as a promising candidate for mitigating AD pathology. This review highlights its potential significance in AD prevention and potentially AD management, particularly given its high bioavailability, capacity to counteract free radicals, and its neuroprotective and chronotherapeutic properties.

However, the incorporation of melatonin into AD treatment will require additional studies. While some animal studies suggest a reduction in Aβ production with melatonin [55, 56], not all findings align with this trend. Lower melatonin doses (1.5 mg/kg/day) in a mouse model of AD (Tg2576) showed no significant modification of brain APP immunoreactivity compared to non-treated animals [165], emphasizing the need to determine efficacious doses. In this context, the consideration of dose-dependent side effects, such as exacerbating breathing difficulties in obstructive sleep apnea patients, impaired blood glucose control when consumed with carbohydrate-rich meals, and an increased risk of nighttime falls due to drowsiness, especially in the elderly, is imperative [236–238]. Challenges, including unsupervised medication use, discrepancies in labeled versus actual melatonin content in supplements, and variations in pharmacokinetics (immediate-release vs. extended-release formulations), underscore the need for physician supervision and strict product regulation [239, 240].

On the positive side, melatonin’s accessibility, affordability, and potential benefits position it as a promising intervention that requires further testing. Studies among both AD dementia populations as well as in preclinical asymptomatic AD coupled with biomarker testing, are needed to address remaining gaps to translation. Since peptides, proteins, and hormones can directly reach the brain when administered intranasally via transport and diffusion along the olfactory and trigeminal nerves [241], future studies should explore whether intranasal melatonin could be a viable therapeutic option for increasing brain melatonin levels in individuals at risk of developing AD. Notably, intranasal melatonin has demonstrated effectiveness in improving sleep in proof-of-concept studies [242].
